# Supplementary material for: PyamilySeq: exposing the fragility of conventional gene (re)clustering and prokaryotic pangenomic inference methods
Source: NAR Genom Bioinform. 2026 Jan 6;8(1):lqaf198. doi: 10.1093/nargab/lqaf198 (PMC12770986; doi:10.1093/nargab/lqaf198)
Supplement: lqaf198_Supplemental_Files [file lqaf198_supplemental_files.zip › PyamilySeq_Supp_3.pdf]

# Supplementary Document
# PyamilySeq: Exposing the fragility of conventional gene (re)clustering and prokaryotic pangenomic inference methods

Nicholas J. Dimonaco [1,2*]

[1]Institute for Global Food Security, School of Biological Sciences, Queen's University Belfast, Belfast, BT9 5DL, UK

[2]Department of Computer Science, Aberystwyth University, Aberystwyth, SY23 3DB, Wales, UK

*To whom correspondence should be addressed. *nicholas@dimonaco.co.uk*

## 1 Supplementary Tables

| **Genome** | **Number of Contigs** | **Number of CDSs** |
|---|---|---|
| Escherichia_coli_110957_gca_000485615.ASM48561v1 | 241 | 4931 |
| Escherichia_coli_1_392_07_s4_c1_gca_000713675.ASM71367v1 | 412 | 5565 |
| Escherichia_coli_5_366_08_s1_c1_gca_000714255.ASM71425v1 | 123 | 5145 |
| Escherichia_coli_b185_gca_000163175.ASM16317v1 | 44 | 4675 |
| Escherichia_coli_536_gca_000013305.ASM1330v1 | 1 | 4540 |
| Escherichia_coli_2362_75_gca_000183005.ASM18300v1 | 83 | 4965 |
| Escherichia_coli_4_0522_gca_000194335.ASM19433v2 | 109 | 5780 |
| Escherichia_coli_2_177_06_s3_c2_gca_000704005.ASM70400v1 | 181 | 5040 |
| Escherichia_coli_113303_gca_000485655.ASM48565v1 | 101 | 4734 |
| Escherichia_coli_113290_gca_000485635.ASM48563v1 | 189 | 4952 |

Supplementary Table 1: The 10 *Escherichia coli* genomes used in 'Pangenomic analysis of 10 *Escherichia coli* genomes reports a consensus of approaches' as reported from Ensembl Bacteria release 59.

| Genome | Number of Contigs | Number of CDSs |
|---|---|---|
| Escherichia_coli_o25b_h4_gca_005671235.ASM567123v1 | 3 | 4966 |
| Escherichia_coli_o25b_h4_gca_005671255.ASM567125v1 | 4 | 5117 |
| Escherichia_coli_o25b_h4_gca_005673435.ASM567343v1 | 5 | 5033 |
| Escherichia_coli_o55_h7_str_cb9615_gca_000025165.ASM2516v1 | 2 | 5146 |
| Escherichia_coli_o7_k1_str_ce10_gca_000227625.ASM22762v1 | 5 | 5140 |
| Escherichia_coli_sms_3_5_gca_000019645.ASM1964v1 | 5 | 4819 |
| Escherichia_coli_umn026_gca_000026325.ASM2632v2 | 3 | 5015 |
| Escherichia_coli_uti89_gca_000013265.ASM1326v1 | 2 | 4837 |
| Escherichia_coli_w_gca_000184185.ASM18418v1 | 3 | 4703 |
| Escherichia_coli_nccp15648_gca_003433275.ASM343327v1 | 4 | 5357 |
| Escherichia_coli_o103_h2_str_12009_gca_000010745.ASM1074v1 | 2 | 5334 |
| Escherichia_coli_o111_nm_gca_005037805.ASM503780v2 | 1 | 4917 |
| Escherichia_coli_o127_h6_gca_900149915.EPEC-E2348_69-V2 | 1 | 4637 |
| Escherichia_coli_o127_h6_str_e2348_69_gca_000026545.ASM2654v1 | 3 | 4872 |
| Escherichia_coli_o157_gca_002208865.ASM220886v2 | 3 | 5498 |
| Escherichia_coli_o157_h7_str_edl933_gca_000006665.ASM666v1 | 2 | 5416 |
| Escherichia_coli_o25b_h4_gca_005670925.ASM567092v1 | 2 | 5171 |
| Escherichia_coli_o25b_h4_gca_005671055.ASM567105v1 | 3 | 4937 |
| Escherichia_coli_gca_900449115.38828_C01 | 4 | 6403 |
| Escherichia_coli_gca_900449225.38891_G01 | 5 | 5180 |
| Escherichia_coli_gca_900449295.39038_F01 | 3 | 6105 |
| Escherichia_coli_gca_900449435.38891_F02 | 4 | 5142 |
| Escherichia_coli_gca_900449455.38891_E02 | 4 | 5217 |
| Escherichia_coli_gca_900449865.39282_C02 | 5 | 7142 |
| Escherichia_coli_gca_900449985.40363_C01 | 5 | 5101 |
| Escherichia_coli_gca_900450185.40105_F02 | 2 | 5436 |
| Escherichia_coli_gca_900520285.ASM90052028v1 | 2 | 4751 |
| Escherichia_coli_gca_900607665.ecoli009 | 5 | 5687 |
| Escherichia_coli_gca_001900535.ASM190053v1 | 3 | 5427 |
| Escherichia_coli_gca_003769125.ASM376912v2 | 3 | 4985 |
| Escherichia_coli_gca_006351885.ASM635188v1 | 5 | 5337 |
| Escherichia_coli_gca_900449095.38891_C01 | 4 | 6494 |
| Escherichia_coli_gca_901733115.Escherichia_coli_MAD | 4 | 4585 |
| Escherichia_coli_o25b_h4_gca_005671075.ASM567107v1 | 2 | 4816 |
| Escherichia_coli_gca_006364695.ASM636469v1 | 4 | 5107 |
| Escherichia_coli_gca_900184875.Unitig | 4 | 4878 |
| Escherichia_coli_gca_900447915.44087_F02 | 3 | 4897 |
| Escherichia_coli_gca_900448265.46338_F02 | 5 | 5212 |
| Escherichia_coli_gca_900448335.50624_F01 | 5 | 5122 |
| Escherichia_coli_gca_900448615.38344_G02 | 2 | 7321 |
| Escherichia_coli_gca_900448835.38389_B01 | 4 | 5222 |
| Escherichia_coli_gca_900448935.38670_B01 | 3 | 5138 |
| Escherichia_coli_gca_900448955.38656_D02 | 4 | 6283 |
| Escherichia_coli_gca_900448985.38490_E02 | 4 | 5584 |
| Escherichia_coli_gca_003790525.ASM379052v1 | 5 | 5914 |
| Escherichia_coli_gca_003991155.ASM399115v1 | 5 | 5304 |
| Escherichia_coli_gca_004011015.ASM401101v1 | 3 | 5202 |
| Escherichia_coli_gca_004114395.ASM411439v1 | 4 | 5057 |
| Escherichia_coli_gca_004358365.ASM435836v1 | 3 | 5666 |
| Escherichia_coli_gca_004564175.ASM456417v1 | 3 | 4993 |
| Escherichia_coli_gca_005890115.ASM589011v1 | 1 | 4474 |
| Escherichia_coli_gca_005954725.ASM595472v1 | 1 | 4484 |
| Escherichia_coli_gca_006337025.ASM633702v1 | 3 | 4933 |
| Escherichia_coli_gca_001902655.ASM190265v1 | 3 | 5297 |
| Escherichia_coli_gca_002011985.ASM201198v1 | 4 | 5100 |
| Escherichia_coli_gca_002156845.ASM215684v1 | 3 | 5513 |
| Escherichia_coli_gca_002164595.ASM216459v2 | 5 | 5052 |
| Escherichia_coli_gca_002554485.ASM255448v1 | 5 | 5240 |
| Escherichia_coli_gca_002846135.ASM284613v1 | 3 | 5418 |
| Escherichia_coli_gca_002860085.ASM286008v1 | 5 | 5176 |
| Escherichia_coli_gca_003122105.ASM312210v1 | 4 | 4930 |
| Escherichia_coli_gca_003571785.ASM357178v1 | 5 | 5129 |
| Escherichia_coli_53638_gca_000167915.ASM16791v2 | 2 | 4924 |
| Escherichia_coli_536_gca_000013305.ASM1330v1 | 1 | 4540 |
| Escherichia_coli_55989_gca_000026245.ASM2624v1 | 1 | 4841 |
| Escherichia_coli_apec_o1_gca_000014845.ASM1484v1 | 3 | 5254 |
| Escherichia_coli_cft073_gca_000007445.ASM744v1 | 1 | 4900 |
| Escherichia_coli_chi7122_gca_000307205.ASM30720v1 | 1 | 4496 |
| Escherichia_coli_gca_000801185.ASM80118v2 | 2 | 4679 |
| Escherichia_coli_gca_001420955.ASM142095v1 | 4 | 5249 |
| Escherichia_coli_gca_001515725.ASM151572v1 | 1 | 4557 |
| Escherichia_coli_gca_001612475.ASM161247v1 | 2 | 5434 |
| Escherichia_coli_gca_001677475.ASM167747v1 | 2 | 5477 |
| Escherichia_coli_gca_001721525.ASM172152v1 | 5 | 5307 |

Supplementary Table 2: The 74 *Escherichia coli* genomes used in 'Pangenomic analysis of 74 *Escherichia coli* genomes reports significant differences based on approach' as reported from Ensembl Bacteria release 59.

| COG Group | PyamilySeq [%] | Roary [%] | Panaroo [%] |
|---|---|---|---|
| ISP | 97 [**26.15**] | 250 [**23.61**] | 548 [**18.72**] |
| CPS | 62 [**16.71**] | 185 [**17.47**] | 586 [**20.02**] |
| MET | 142 [**38.27**] | 410 [**38.71**] | 1,298 [**44.34**] |
| PC | 70 [**18.86**] | 214 [**20.21**] | 495 [**16.91**] |

Supplementary Table 3: Reported here are the broad functional COG categories in raw numbers and proportions. The COG group acronyms are as follows: ISP - INFORMATION STORAGE AND PROCESSING, CPS - CELLULAR PROCESSES AND SIGNALING, MET - METABOLISM and PC - POORLY CHARACTERIZED.

| Groups | 1 CPU | | | 8 CPU | | |
|---|---|---|---|---|---|---|
| | 8,000 MB | 4,000 MB | 800 MB | 8,000 MB | 4,000 MB | 800 MB |
| Core genes: | 1952 | 1952 | 1953 | 1952 | 1952 | 1953 |
| Soft core genes: | 1160 | 1160 | 1160 | 1160 | 1160 | 1160 |
| Shell genes: | 2644 | 2644 | 2640 | 2644 | 2644 | 2640 |
| Cloud genes: | 22455 | 22455 | 22458 | 22455 | 22455 | 22458 |
| Total: | 28211 | 28211 | 28211 | 28211 | 28211 | 28211 |

Supplementary Table 4: The different clustering results we get for the number of core genes due to the different CPU and Memory allocations.

| CD-HIT initialisations | 1 CPU | | | 8 CPU | | |
|---|---|---|---|---|---|---|
| | 8,000 MB | 4,000 MB | 800 MB | 8,000 MB | 4,000 MB | 800 MB |
| Buffer (**MB**) | 22 | 22 | 22 | 176 | 176 | 176 |
| Table (**MB**) | 71 | 71 | 71 | 143 | 143 | 143 |
| Total (**MB**) | 283 | 283 | 283 | 509 | 509 | 509 |
| Representatives | 40,000,000 | 40,000,000 | 1,054,982 | 40,000,000 | 40,000,000 | 593,077 |
| Word Count Entries | 964,607,788 | 464,607,788 | 64,607,788 | 936,320,494 | 436,320,494 | 36,320,494 |

Supplementary Table 5: The different initialisation setups that CD-HIT reports based on different CPU and memory allocations.

| Groups | Roary 10 *E. coli* genomes | | | Roary 74 *E. coli* genomes | | |
|---|---|---|---|---|---|---|
| | No-Align | MAFFT | PRANK | No-Align | MAFFT | PRANK |
| Core genes | 3,078 | 3,078 | 3,077 | 1,023 | 1,026 | 1023 |
| Soft core genes | 0 | 0 | 0 | 872 | 848 | 866 |
| Shell genes | 3,470 | 3,463 | 3,458 | 4,740 | 4,763 | 4,709 |
| Cloud genes | 6,172 | 6,181 | 6,187 | 31,709 | 31,714 | 31,799 |
| Total genes | 12,720 | 12,722 | 12,722 | 38,344 | 38,351 | 38,397 |

Supplementary Table 6: The influence that the **align core genes** option with PRANK (`-e`) or MAFFT `-e -n`) has on the output of Roary while all other parameters are set to default and the CPU allocation is fixed to 8.

| Sequence | Pos 1 | Pos 2 | Pos 3 | Pos 4 | Pos 5 | Pos 6 | Pos 7 | Pos 8 | Pos 9 | Pos 10 | .. | Pos 20 |
|---|---|---|---|---|---|---|---|---|---|---|---|---|
| DNA Seq 1 | ATG | GCT | TCT | ACT | CAG | CGT | ATT | CTG | GTG | GGT | .. | TGA |
| DNA Seq 2 | ATG | GCC | AGC | ACA | CAA | AGA | ATA | TTA | GTA | GGA | .. | TAG |
| AA Seq | M | A | S | T | Q | R | I | L | V | G | .. | * |

Supplementary Table 7: An example of where two different nucleotide sequences can encode the same amino acid sequence using the same codon table (11). DNA column codes for the same amino acid sequence, despite different nucleotide sequences.

| Tool | Normalised Robinson-Foulds | Standard Deviation |
|---|---|---|
| Roary | 0.8920 | 0.0695 |
| Roary_dsp | 0.9080 | 0.0683 |
| Panroo | 0.9120 | 0.0682 |
| Panroo_refound | 0.8937 | 0.1597 |
| PyamilySeq_90_80 | 0.9108 | 0.0732 |
| PyamilySeq_90_0 | 0.8956 | 0.0708 |

Supplementary Table 8: Reported here are the normalised all-against-all Robinson-Foulds distances computed from the single gene trees from each of the tools studied. Roary_dsp stands for Roary performed with the **`don't split paralogs`** option, and Panaroo_refound stands for allowing refound sequences to be processed as annotated sequences. PyamilySeq_90_80 and PyamilySeq_90_0 stand for when PyamilySeq was either run with 80% or 0% `length difference cutoff`.

| **Gene Groups** | **80% / [0%]** |
|---|---|
| First_core_99 | 357 / [**1953**] |
| First_core_95 | 2139 / [**1162**] |
| First_core_15 | 3219 / [**2633**] |
| First_core_0 | 37014 / [**22463**] |
| extended_core_99 | 0 / [**28**] |
| extended_core_95 | 17 / [**31**] |
| extended_core_15 | 95 / [**217**] |
| extended_core_0 | 264 / [**401**] |
| combined_core_99 | 0 / [**3**] |
| combined_core_95 | 0 / [**0**] |
| combined_core_15 | 8 / [**7**] |
| combined_core_0 | 4 / [**2**] |
| Second_core_99 | 0 / [**0**] |
| Second_core_95 | 1 / [**3**] |
| Second_core_15 | 41 / [**76**] |
| Second_core_0 | 356 / [**1006**] |
| only_Second_core_99 | 136 / [**149**] |
| only_Second_core_95 | 494 / [**523**] |
| only_Second_core_15 | 4030 / [**3948**] |
| only_Second_core_0 | 38443 / [**36133**] |
| # FCs (Incl. Singletons) | 42730 / [**28211**] |
| # SCs (Incl. Singletons) | 85824 / [**68954**] |
| # FCs (w/ Add' Second Seqs, No New Genomes) | 17 / [**404**] |

Supplementary Table 9: Detailed summary of gene family/group classifications reported by reclustering StORF sequences with PyamilySeq under two parameter sets: 80% and 0% `length difference cutoff` (values in square brackets). Each row represents a category of gene families based on their occurrence and relationship across the original (First) and newly added (Second) sequences. The final three rows summarise total gene family counts: # FCs (Incl. Singletons): Total number of First gene families including singletons. # SCs (Incl. Singletons): Total number of Second gene families including singletons. # FCs (w/ Add'l Second Seqs, No New Genomes): Number of First_core families that were expanded by additional Second sequences, but did not include any newly added genomes.

## 2 Supplementary Figures

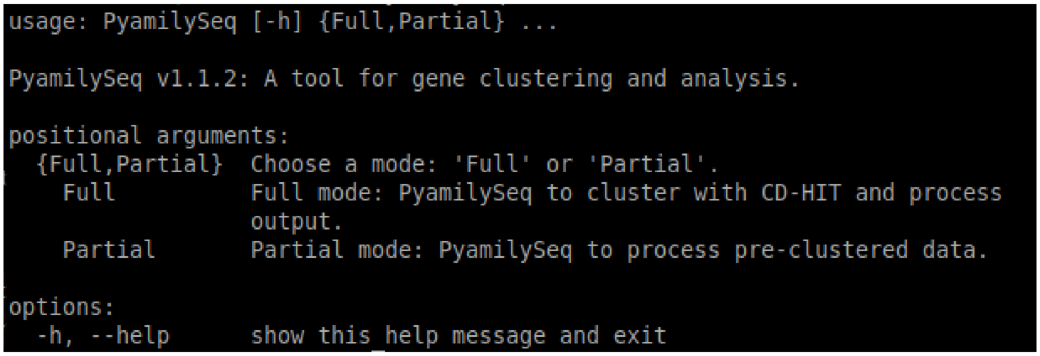


Supplementary Figure 1: The PyamilySeq menu system showing the two main modes of operation - Full and Partial.

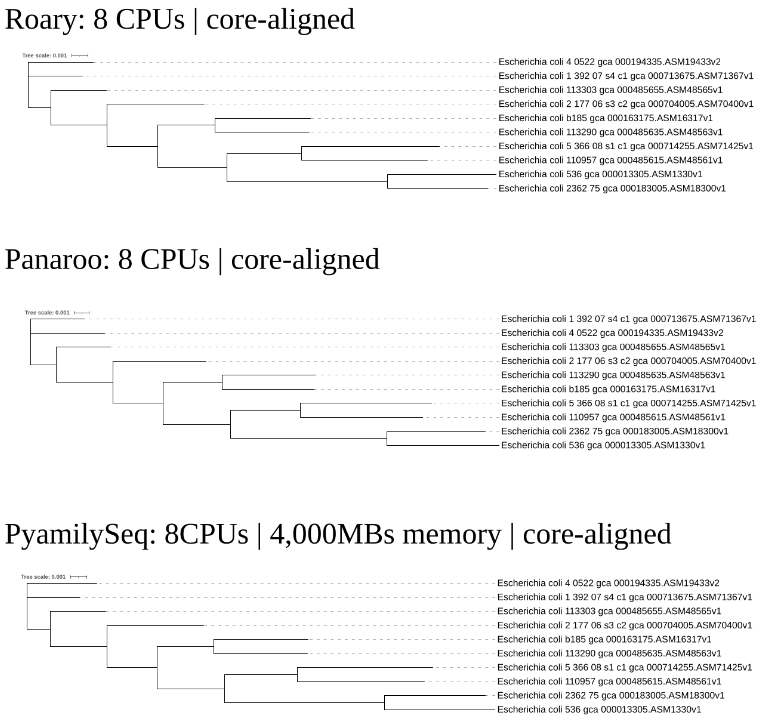


Supplementary Figure 2: The three phylogenetic trees built using the ‘core-gene’ families identified by Roary, Panaroo and PyamilySeq.

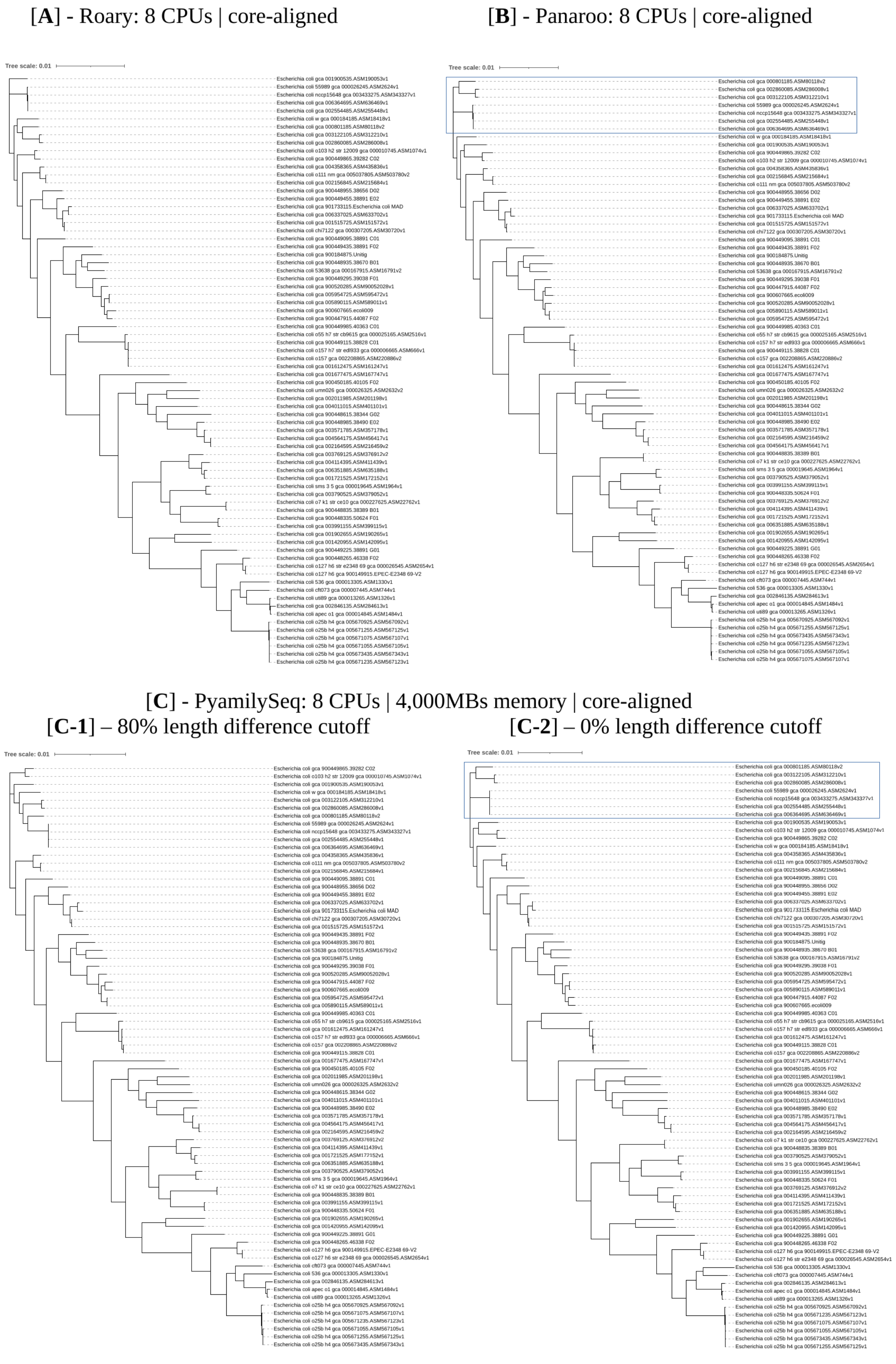


Supplementary Figure 3: The three phylogenetic trees built using the 'core-gene' families identified by Roary (A), Panaroo (B) and PyamilySeq[AA] (C) for the 74 genomes.

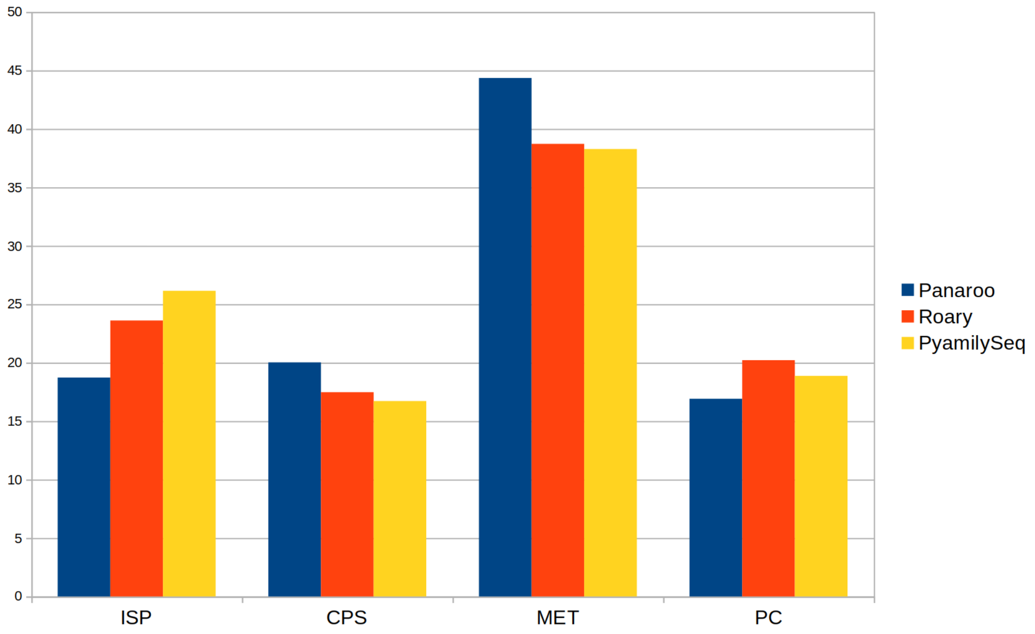


Supplementary Figure 4: Broad functional overview from the core gene sets reported by PyamilySeq, Roary and Panaroo using COG categories from eggNOG-mapper. The COG group acronyms are as follows: ISP - INFORMATION STORAGE AND PROCESSING, CPS - CELLULAR PROCESSES AND SIGNALING, MET - METABOLISM and PC - POORLY CHARACTERIZED.

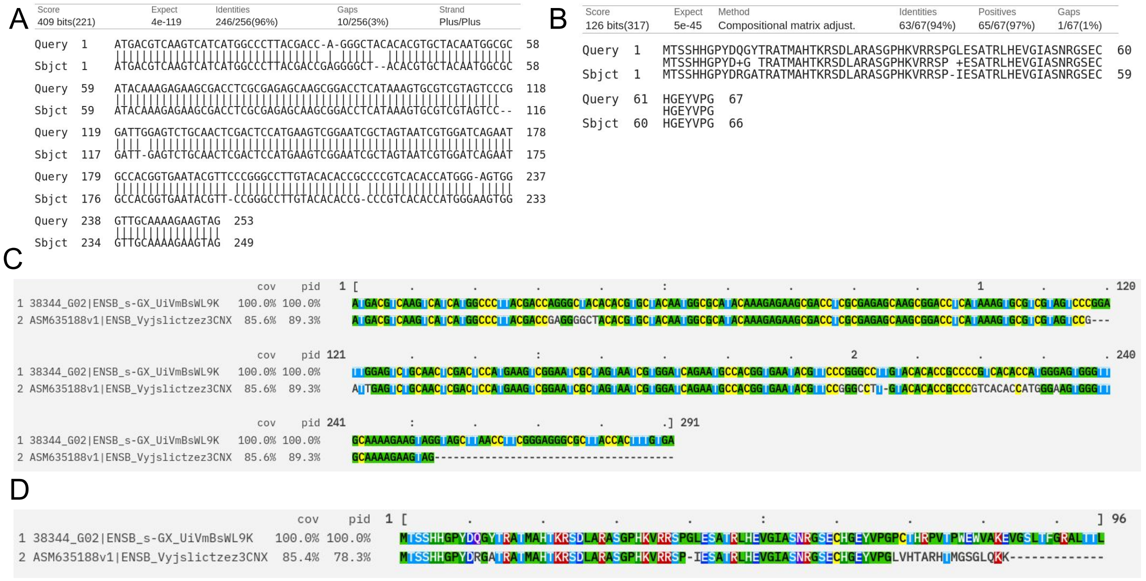


Supplementary Figure 5: Panels depicting some of the problems with clustering in the DNA and AA spaces and how we get different results from different methods. Panels **A** and **B** report the BLAST alignments of sequences *ENSB_s-GX_UiVmBsWL9K* and *ENSB_Vyjslictzez3CNX* in DNA and AA, respectively, where both reported a higher than 90% identity. Panels **C** and **D** report the multiple sequence alignment conducted by Clustal Omega in DNA and AA respectively but reporting a percentage identity of less than 90%.

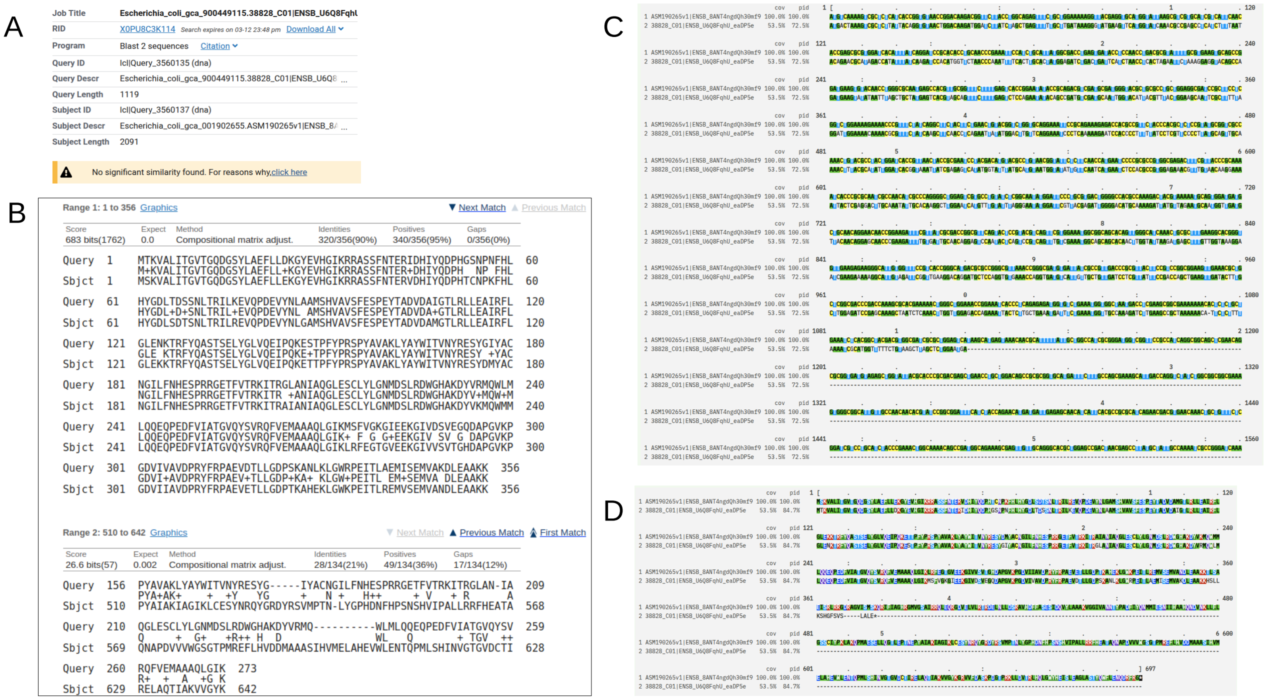


Supplementary Figure 6: Panels reporting BLAST and Clustal-Omega alignment results for *ENSB_U6Q8FqhU_eaDP5e* and *ENSB_8ANT4ngdQh30mf9*. Panels **A** and **B** report how 'no significant similarity' was found by BLAST in the AA alignment, but two segments were able to be aligned in the DNA alignment, the first with 90% identity. Panels **C** and **D** shows the Clustal-Omega alignments, which were reported at 72.5% and 84.7% for DNA and AA, respectively.

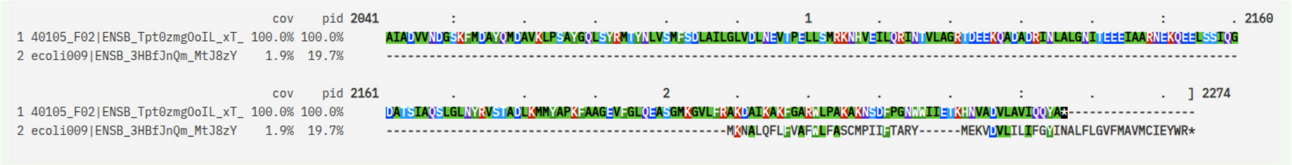


Supplementary Figure 7: Alignment of two sequences from Roary's group_9 that have almost no similarity but nonetheless were grouped together into the same gene family with the `-s dont split paralogs` options.

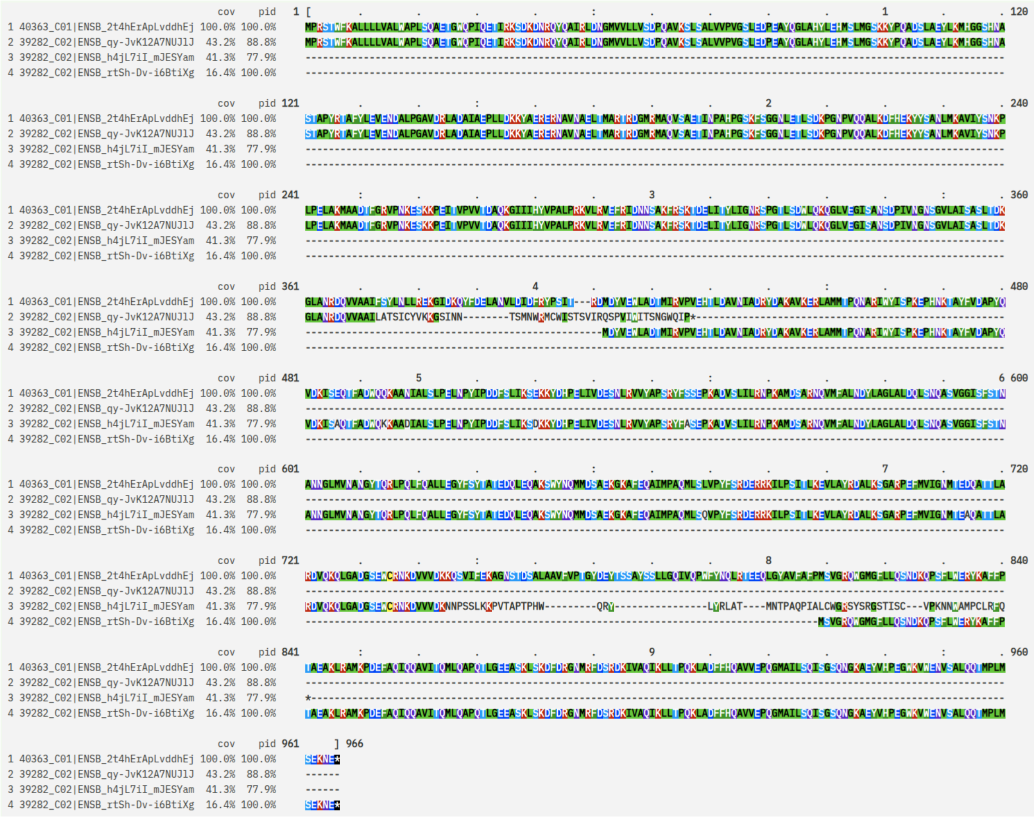

Supplementary Figure 8: Alignment of sequences in Panaroo's group_21184, showing a 962 AA sequence and three shorter sequences aligning to different regions of the same *E. coli* pitrilysin protein.

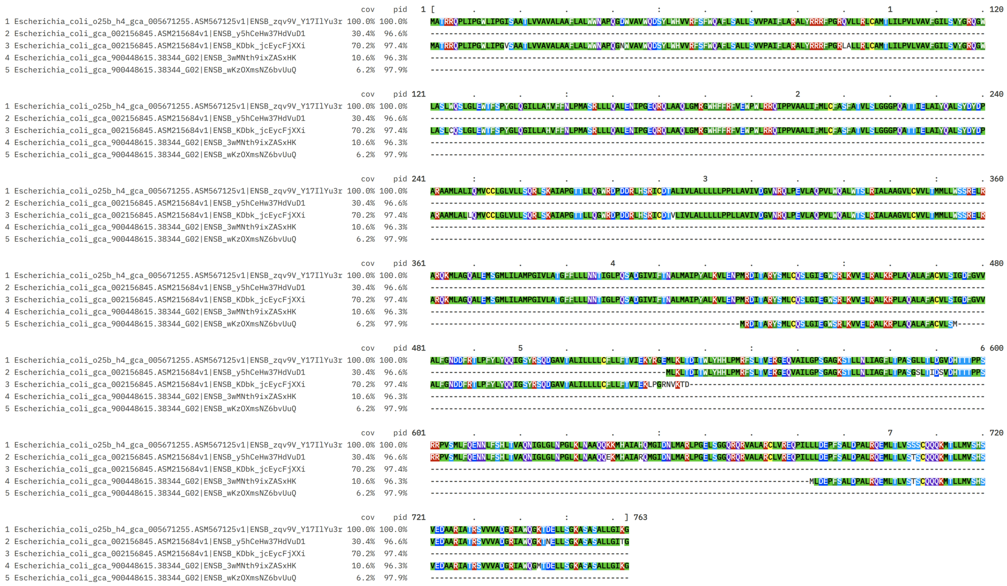

Supplementary Figure 9: Alignment of sequences from group_21164. The first sequence represents the long gene from the outlier genome, followed by two pairs of shorter sequences (699 nt and 1,611 nt) and another smaller pair of (246 and 144 nt).

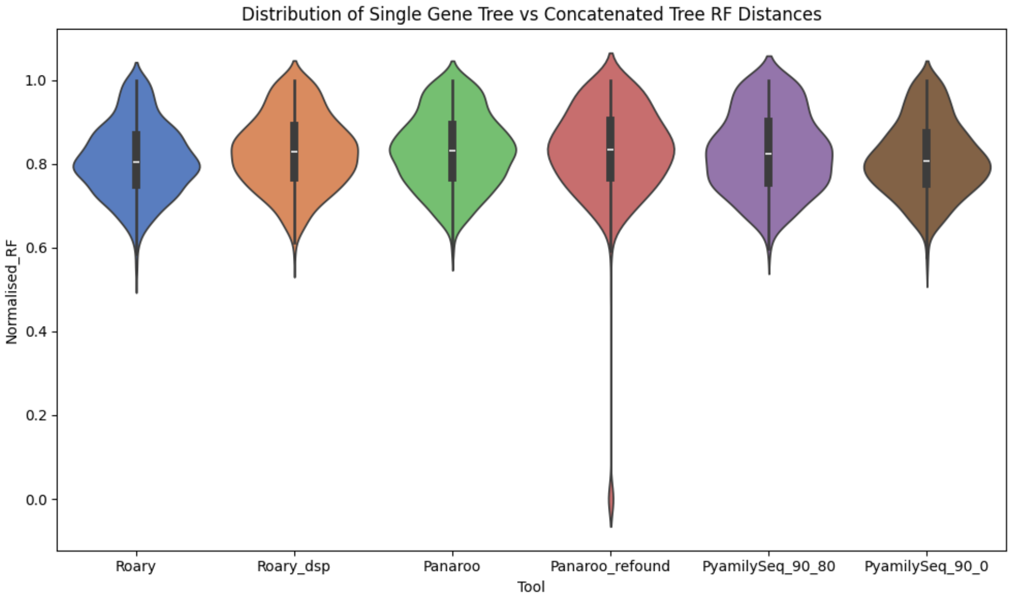


Supplementary Figure 10: The normalised Robinson–Foulds distances computed for each tool's set of core single gene trees against the concatenated gene trees. Roary_dsp stands for Roary performed with the `don't split paralogs` option, and Panaroo_refound stands for allowing refound sequences to be processed as annotated sequences.

# 3 Supplementary Listings

- **First_core_#**: (First Core) Gene groups identified in the **initial round of clustering** before any reclustering is performed - These results are directly comparable to Roary and Panaroo.
- **extended_core_#**: Gene groups from the initial clustering that were **expanded** by incorporating sequences from the reclustered dataset during the second round of clustering.
- **combined_core_#**: Gene groups where sequences from the reclustered dataset **merged two or more original clusters** into a single, larger group.
- **Second_core_#**: (Second Core) New gene groups that are classified as core **exclusively from the reclustered sequences**, without contributions from the initial clustering - First round sequences may be present but are not counted.
- **only_Second_core_#**: A subset of **Second_core_#** containing gene groups that consist **entirely of reclustered sequences**, with no overlap with the initial dataset.

List 1: Summary of the gene group classifications in PyamilySeq output. The notation distinguishing between **First Core (FC)** - results from the initial clustering, and **Second Core (SC)** - results from reclustering. The # symbol denotes the percentage identity thresholds used for defining gene groups, with default values set to 99%, 95%, 15%, and 0%.

Listing 1: CD-HIT initialisation with PyamilySeq Full mode default parameters (-T 8 and -M 4000)

```
total seq: 50325
longest and shortest : 6925 and 29
Total letters: 15353668
Sequences have been sorted
Approximated minimal memory consumption:
Sequence        : 24M
Buffer          : 8 X 12M = 100M
Table           : 2 X 66M = 132M
Miscellaneous   : 0M
Total           : 258M
Table limit with the given memory limit:
Max number of representatives: 4000000
Max number of word counting entries: 467728268
```

Listing 2: CD-HIT initialisation using 8 CPU threads but CD-HITs default memory (-T 8 and -M 800)

```
total seq: 50325
longest and shortest : 6925 and 29
Total letters: 15353668
Sequences have been sorted
Approximated minimal memory consumption:
Sequence        : 24M
Buffer          : 8 X 12M = 100M
Table           : 2 X 66M = 132M
Miscellaneous   : 0M
Total           : 258M
Table limit with the given memory limit:
Max number of representatives: 1090357
Max number of word counting entries: 67728268
```

Listing 3: CD-HIT initialisation using CD-HITs default parameters (-T 1 -M 800)

```
total seq: 50325
longest and shortest : 6925 and 29
Total letters: 15353668
Sequences have been sorted
Approximated minimal memory consumption:
Sequence        : 24M
Buffer          : 1 X 12M = 12M
Table           : 1 X 66M = 66M
Miscellaneous   : 0M
Total           : 104M
Table limit with the given memory limit:
Max number of representatives: 1400522
Max number of word counting entries: 86994327
```

# 4 Software versions used

```
PyamilySeq: v1.2.0
CD-HIT: v4.8.1
Panaroo: v1.5.1
Roary: v3.13.0
MAFFT: v7.490
FastTree: v2.1.11
ETE 3: v3.1.3
BLAST: 2.16.0
clustalo: v1.2.4
mview: v1.67
```

# 5 Processing

## 5.1 Input handling with Seq-Combiner

A major hurdle in bioinformatic analysis is file format compatibility. As highlighted in my previous work on the ORForise genome annotation comparison platform [1], each annotation tool delivers its own output format, and even tools pertaining to use the General Feature Format (GFF), often have tool-specific interpretations. Tools that process genome annotation output, frequently struggle with these varying formats, requiring users to modify annotations to meet specific input requirements. For example, Roary and Panaroo require their input to be Prokka-formatted [2] GFF files.

To address these compatibility issues, the PyamilySeq toolkit includes `Seq-Combiner`, which generates compatibility-assured input files for PyamilySeq. Seq-Combiner takes a directory of matching genome FASTA and GFF files or GFF files with '##FASTA - Seq' appended to the bottom and prepends the file name to each gene ID. Additionally, to support the use of other sequence clustering techniques, Seq-Combiner can process pre-extracted FASTA files and their sequences into the required naming convention, ensuring they can be seamlessly used within PyamilySeq. This flexibility ensures compatibility with diverse input data formats generated by various genome annotation and alignment tools.

An example of the sequence renaming process, which uses the names of the GFF/FASTA files, is shown below:

Listing 4: Seq-Combiner Sequence renaming.

```
GFF/FASTA Filename: Escherichia_coli..[.gff/.fasta]
GeneID: E1CxwDWD5uDlWpi

>Escherichia_coli..|E1CxwDWD5uDlWpi
>Escherichia - [GenusID]
>Escherichia_coli.. - [SpeciesID/GenomeID]
>Escherichia_coli..|E1CxwDWD5uDlWpi - [GeneID]
```

## 5.2 Group summaries and investigation

The Group-Summary tool provides a detailed overview of CD-HIT clustering results, helping researchers evaluate and optimise clustering parameters. By analysing gene clustering patterns across genomes, it enables users to observe how parameter selection can affect results, such as the number of gene families, the distribution of core and accessory genes, and the presence of multicopy gene clusters.

Group-Summary processes the CD-HIT .clstr files and extracts key statistics for each cluster. The tool calculates and outputs comprehensive summaries for each cluster, including: The number of sequences in the cluster, The number of Genomes that the sequences are present in, Average sequence length, Length range (minimum to maximum), Average percentage identity, Identity range (minimum to maximum), The number of genomes with $\geq$1 gene and the percentage of genomes with $\geq$ gene.

A key feature of Group-Summary is its ability to assess multicopy genes, identifying clusters where genomes contain multiple representatives. It categorises these by predefined percentage ranges (e.g., 20–40%, 40–60%), providing insights into gene duplication and paralogy. This categorisation helps determine whether clustering parameters accurately reflect dataset biology, informing decisions on core, accessory, or duplicated gene families.

Additionally, PyamilySeq includes Group-Splitter, which applies Levenshtein distance [3] to refine gene clusters. This tool was used in this study to better decipher the impacts of clustering limitations and sequence divergence in the reported gene families.

# References


[1] Nicholas J Dimonaco, Wayne Aubrey, Kim Kenobi, Amanda Clare, and Christopher J Creevey. No one tool to rule them all: prokaryotic gene prediction tool annotations are highly dependent on the organism of study. *Bioinformatics*, 38(5):1198–1207, 2022.

[2] Torsten Seemann. Prokka: rapid prokaryotic genome annotation. *Bioinformatics*, 30(14):2068–2069, 2014.

[3] Vladimir I Levenshtein et al. Binary codes capable of correcting deletions, insertions, and reversals. In *Soviet physics doklady*, volume 10, pages 707–710. Soviet Union, 1966.
